# The Diagnosis of Trachoma

**Published:** 1907-08-31

**Authors:** 


### OPHTHALMOLOGY.

### THE DIAGNOSIS OF TRACHOMA.

Considerable variety is seen in the onset and course of trachoma. Some cases are comparatively mild, others more or less severe. Four classes may be distinguished: first, "suspicious cases," in which a definite diagnosis cannot be made until the case has been under observation for some time; second, "mild cases," in which there is only a moderate hypertrophy of the conjunctival papillæ, no follicles in the tarsal conjunctiva, and only a few in the retrotarsal folds, and no secretion; third, "moderately severe cases," in which there is considerable swelling in the upper fornices, many follicles on both lids and in the tarsal conjunctiva, and more or less profuse secretion; fourth, "severe cases," in which sequelæ are evident, such as pannus, corneal ulcer, leucoma, anterior staphyloma, scarring, entropion, trichiasis, or xerophthalmos.

It is in the first two classes that there is greatest difficulty in making a diagnosis; in the other two the disease is more or less evident. In the initial stage trachoma is not infrequently overlooked or wrongly diagnosed, so it is most important to make a thorough and careful examination of the whole conjunctival sac. The lower lid is easily examined; the upper one should be well everted, and, if possible, a second eversion made, for it is clinically established that the formation of the trachomatous follicles is for a long time confined to the retrotarsal folds. The essential feature of all types of trachoma is the trachoma follicle. This first appears as a small greyish-yellow spot, about the size of a pin's head, in the deeper part of the conjunctiva, usually without any sign of inflammation. The small spot gradually develops into a round, projecting, large, and coarse follicle, grey or yellowish red in colour. Microscopically the trachoma granulation is essentially the same as the follicle of follicular conjunctivitis, but clinically the two diseases are distinct, though their differential diagnosis presents some difficulty. The chief points of difference are:—

| *Trachoma.* | *Follicular Conjunctivitis.* |
| --- | --- |
| Occurs at all ages, but mostly in adults, and is seldom met with in the upper classes. | Mostly found in the young and of all classes. |
| Caused by a specific virus, and, as far as is known at present, arises only through infection with the conjunctival discharge from a case of trachoma. | A "folliculosis," or swelling of the conjunctival follicles, is produced by various irritants, such as dust, vitiated air, atropine or eserine. This renders the eye more liable to catarrhal infection, and may give rise to follicular conjunctivitis, especially when the general tone of the patient is low, but never to trachoma. Follicular swelling is sometimes associated with eye-strain and with simple catarrhal conjunctivitis. |
| Trachoma follicles lie deep in the tissues, and are frequently confluent. | The follicles seem to be on the conjunctiva rather than in it, and have no tendency to spread. |
| They are found on both lids and on the tarsal conjunctiva. | Usually confined to the lower lid, and never affect the tarsal conjunctiva. |
| They are round, large, and coarse, grey, or yellowish red, translucent, with ill-defined outline. | Often horizontally oval, smaller, transparent, glassy appearance, with well-defined outline. |
| There is little or no regularity in their arrangement. | More regular. |
| The conjunctival connective tissue becomes affected, and soon the conjunctiva loses its transparency, becomes thickened, congested, and rough, owing to the swelling of the papillæ, and frequently the retrotarsal folds become much hypertrophied. | Here the conjunctiva is never seriously affected, remaining transparent, smooth, and often not congested. |
| The trachoma follicles ultimately undergo definite and characteristic changes, resulting in cicatrisation with various sequelæ, as pannus, etc. | The follicles, after a longer or shorter period, disappear, and never lead to scarring, so leaving no permanent injury to the mucous membrane. |

Cases of "acute trachoma" are rare; acute symptoms are oftenest due to the infection of an already trachomatous conjunctiva by some organism producing a muco-purulent or purulent conjunctivitis. In these cases the hypertrophy of the papillæ is so great that the trachoma bodies are obscured, and an accurate diagnosis is impossible until the acute symptoms have subsided. A bacteriological examination should be made. Trachoma is a very chronic disease, generally attacking both eyes, and there is frequently a long latent period without any marked subjective symptoms. Gradually the trachoma bodies give rise to more or less inflammatory reaction, with hypertrophy of the papillæ and purulent discharge. When trachoma has lasted some time the thickening of the conjunctiva or infiltration of the tarsus produces a very characteristic ptosis, which should prompt one to examine carefully the whole conjunctival sac. The diffuse infiltration of the adenoid tissue produces a gelatinous change in the conjunctiva, called "Stellwag's brawny œdema"; when this occurs it is a very definite diagnostic feature.

In the later stages the diagnosis is much easier, and is absolutely certain when the conjunctiva becomes gelatinous, with pannus and scarring supervening. Typical pannus is strong evidence of trachoma, but is not a constant symptom. A somewhat similar condition of the cornea is seen in phlyctenular affections of the cornea and in scrofulous marginal keratitis. These conditions begin in various parts of the cornea, and there are no granulations and no scars in the conjunctiva; under general and local treatment they rapidly improve.

Trachoma pannus almost invariably begins as a crescent at the upper margin of the cornea, spreading gradually towards the centre. When a few misdirected cilia rub on the cornea a superficial change is produced resembling mild pannus; a large healing vascular ulcer presents sometimes a somewhat similar picture. Careful examination of the everted lid should establish a diagnosis. Scarring is an important sign of trachoma. This produces a symblepharon posterius, or a shortening of the retrotarsal folds, and incurving of the upper tarsus, going on to produce entropion and trichiasis.

Scarring of the conjunctiva is also produced by mechanical and chemical injuries; then it is usually unilateral. Cicatrisation also follows diphtheritic and gonorrhœal conjunctivitis, and pemphigus: in these there is no pannus, and the history is an aid to the diagnosis.

